# Modeling creative abduction Bayesian style

**DOI:** 10.1007/s13194-018-0234-4

**Published:** 2018-11-03

**Authors:** Christian J. Feldbacher-Escamilla, Alexander Gebharter

**Affiliations:** 1 Duesseldorf Center for Logic and Philosophy of Science (DCLPS), University of Duesseldorf, Universitaetsstrasse 1, 40225 Duesseldorf, Germany; 2 Department of Theoretical Philosophy, Faculty of Philosophy, University of Groningen, Oude Boteringestraat 52, 9712 GL Groningen, Netherlands

**Keywords:** Creative abduction, Theoretical concepts, Bayes nets, Unification, Novel predictions, Underdetermination

## Abstract

Schurz (Synthese 164:201–234, 2008) proposed a justification of creative abduction on the basis of the Reichenbachian principle of the common cause. In this paper we take up the idea of combining creative abduction with causal principles and model instances of successful creative abduction within a Bayes net framework. We identify necessary conditions for such inferences and investigate their unificatory power. We also sketch several interesting applications of modeling creative abduction Bayesian style. In particular, we discuss use-novel predictions, confirmation, and the problem of underdetermination in the context of abductive inferences.

## Introduction

One can basically distinguish two kinds of abductive inferences: those generating new hypotheses and those aiming at determining the best hypothesis from a set of available candidates. Let us call abductive inferences of the former kind *creative*, and those of the latter kind *selective*.^1^ While most of the philosophical literature on abduction focuses on selective abduction (see, e.g., Lipton 2004; Niiniluoto 1999; Williamson 2016), there is also an increasing interest in creative abduction (cf. Douven 2017).

In contrast to selective abduction and other kinds of inferences (such as deduction and induction), creative abduction is intended as an inference method for generating hypotheses featuring new theoretical concepts on the basis of empirical phenomena. Most philosophers of science are quite sceptical about whether a general approach toward such a *logic of scientific inquiry* can be fruitful. However, since theoretical concepts are intimately connected to empirical phenomena via dispositions (see, e.g., Carnap 1936, 1937), a restriction of the domain of application of such an approach to empirically correlated dispositions might be promising. Schurz (2008) differentiates between different patterns of abduction and argues for the view that at least one kind of creative abduction can be theoretically justified. In a nutshell, his approach is based on the idea that inferences to theoretical concepts unifying empirical correlations among dispositions can be justified by Reichenbach’s (1956) principle of the common cause.

In this paper we take up Schurz’ (2008) proposal to combine creative abduction and principles of causation. We model cases of successful creative abduction within a Bayes net framework which can, if causally interpreted, be seen as a generalization of Reichenbach’s (1956) ideas (cf. Glymour et al. 1991). Such a move allows us to specify general conditions which have to be satisfied in order to generate hypotheses involving new theoretical concepts and to describe their unificatory power in a more fine-grained way. In addition, it can be used to shed new light on several other issues discussed within philosophy of science. In this paper we will sketch how it allows for handling cases in which we can only measure non-strict (i.e., probabilistic) empirical dependencies among dispositions, and how it paves the way for new applications to other topics within philosophy of science. We consider our analysis of successful instances of creative abduction by means of Bayes net models as another step toward a unified Bayesian philosophy of science in the sense of Sprenger and Hartmann (forthcoming).

The paper is structured as follows: In Section 2 we introduce Schurz’ (2008) approach to creative abduction. We also explain how it allows for unifying strict empirical correlations among dispositions and how it can be justified by Reichenbach’s (1956) principle of the common cause. In Section 3 we then briefly introduce the Bayes net formalism, present our proposal how to model successful cases of creative abduction within this particular framework, and identify necessary conditions for such cases. Next we investigate the unificatory power gained by creative abduction in the Bayesian setting and draw a comparison with the unificatory power creative abduction provides in the strict setting. In Section 4 we sketch possible applications of our analysis to other topics within philosophy of science. In particular, we discuss the generation of use-novel predictions, new possible ways of applying Bayesian confirmation theory, a possible (partial) solution to the problem of underdetermination, and the connection of modeling successful instances of creative abduction Bayesian style to epistemic challenges tackled in the causal inference literature. We conclude in Section 5.

## Creative abduction, unification, and the principle of the common cause

In this section we present Schurz’ (2008) approach to creative abduction. Following Schurz, we focus on a simple analysis of dispositions as introduced by the early logical empiricists (e.g., Carnap 1936, 1937).^2^ According to this analysis, whether an object *x* has a disposition *D* depends on whether certain test conditions *T* lead to a specific reaction *R*. For an object *x* to be soluble in water, for example, it is required that *x* dissolves at some time *t* if put into water at *t*:

1 $$ \forall t(T(x,t)\rightarrow (D(x)\leftrightarrow R(x,t))) $$

According to the traditional understanding, *T* and *R* are empirical concepts, while the dispositional concept *D* is a not directly observable theoretical concept. Note that Eq. 1 comes close to a partial definition of *D* on the basis of *T* and *R*, except that the dispositional term is not relativized to *t*. What distinguishes the characterization of a disposition *D*(*x*) as provided in Eq. 1 from a purely conventional definition of a disposition with reference to time (e.g., by replacing *D*(*x*) with *D*(*x*,*t*) in Eq. 1, where *D*(*x*,*t*) might be interpreted as *x* is soluble in water at some point in time *t*) is that Eq. 1 is empirically creative in the sense that it allows for deducing empirical statements which cannot be deduced from our background postulates on statements containing *T* and *R* alone. It is a well-known fact that the only non-conservative (or creative) import of Eq. 1 is the following assumption about the uniformity of test-reaction pairs: If at some time *t* an object *x* satisfies the test conditions and brings about the corresponding reaction, then *x* will do so at any time *t*:

2 $$ \exists t(T(x,t)\wedge R(x,t))\rightarrow \forall t(T(x,t)\rightarrow R(x,t)) $$

Equations 1 and 2 are empirically equivalent, where two statements “are empirically equivalent just in case they have the same class of empirical, viz., observational, consequences [and …] the empirical consequences of any statement are those of its logical consequences formulable in an observation language” (Laudan and Leplin 1991, p. 451; cf. also Okasha 1997, p. 251). That the empirical content of Eq. 2 is implied by Eq. 1 is straightforward, since Eq. 2 contains only (logical and) empirical expressions and is a direct consequence of Eq. 1. That all statements containing only (logical and) empirical expressions that are consequences of Eq. 1 can be deduced already from Eq. 2 can be shown by definition theoretical means (cf. Essler and Trapp^1978^).

If Eq. 2 has been established on empirical grounds, then introducing a disposition *D* via Eq. 1 is a theoretical means to explain Eq. 2. However, not much is gained by introducing *D* since for each regularity among test-reaction pairs a distinct disposition has to be postulated. Things become more interesting once we focus on regularities among several dispositions *D*_1_,...,*D*_*n*_, each characterized by a corresponding test-reaction pair consisting of *T*_*i*_ and *R*_*i*_ (with 1 ≤ *i* ≤ *n*). Now assume that we found strict pairwise empirical correlations between all of these dispositions *D*_1_,...,*D*_*n*_, meaning that

3 $$ D_{i}(x)\leftrightarrow D_{i + 1}(x)\text{ for all } 1\leq i<n. $$

This amounts to the assumption that the following statement has been empirically established:

4 $$ \exists t(T_{i}(x,t)\wedge R_{i}(x,t))\rightarrow \forall t(T_{j}(x,t)\rightarrow R_{j}(x,t))\text{ for all \(1\leq i,j\leq n\)} $$

Let us call each statement of this form a *crossed uniformity assumption*. Given *n* test-reaction pairs for *n* dispositions *D*_1_,...,*D*_*n*_, we get *n*^2^ such crossed uniformity assumptions (Schurz 2008, p. 226). It is a logical fact that this is empirically equivalent to introducing one higher-level dispositional concept $\mathcal {D}$ characterized by *n* test-reaction pairs:

5 $$ \forall t(T_{i}(x,t)\rightarrow (\mathcal{D}(x)\leftrightarrow R_{i}(x,t)))\text{ for all \(1\leq i\leq n\)} $$

Note that introducing the theoretical concept $\mathcal {D}$ via Eq. 5 reduces the number of law statements from *n*^2^ to *n*. In this sense such a reduction can be understood as unificatory. The abductive inference consists in the introduction of $\mathcal {D}$ via Eq. 5 on the basis of Eq. 4. It can be illustrated on the following example inspired by Hempel (1965): Assume that at some time the inhabitants of a not too distant possible world realized that some objects have the disposition to attract iron (*D*_1_) and that some objects have the disposition to produce electricity when moved along a wire (*D*_2_), meaning that they introduced the two theoretical concepts *D*_1_ and *D*_2_ on the basis of Eq. 2 and in accordance with Eq. 1. Suppose further that both discoveries were made independently of each other, but that people found out later on that the dispositions *D*_1_ and *D*_2_ are correlated (Eq. 3) via observing that their corresponding test and reaction conditions coincided (Eq. 4). They might then have started to explain this correlation by introducing the higher-level disposition of generating an electromagnetic field $\mathcal {D}$ via Eq. 5.

Note that creative abduction as discussed above can be interpreted either in a realist or an instrumentalist way. Under the latter interpretation $\mathcal {D}$ is taken to be nothing over and above a more or less useful theoretical means to unify empirical descriptions of certain phenomena of interest that can—in principle—be replaced by any other concept with equal empirical adequacy and unificatory power. Under the realist interpretation, on the other hand, $\mathcal {D}$ is assumed to represent a real structure; statements involving $\mathcal {D}$ are considered to be either true or false. Schurz (2008) made a strong case in favour of a realist interpretation by endorsing Reichenbach’s (1956) common cause principle:

**(CCP)**: If two properties *A* and *B* are correlated and neither *A* causes *B* nor *B* causes *A*, then *A* and *B* are effects of a common cause *C*.

**(CCP)** demands that every correlation among any pair of properties not standing in direct causal dependence to each other has to be explained by the existence of an independent common cause. In this sense **(CCP)** provides a way of causally unifying observed regularities. In the case of pairwise empirically correlated dispositions such as *D*_1_,...,*D*_*n*_ above, **(CCP)** supports a realist interpretation of the unifying higher-level disposition $\mathcal {D}$: The correlation among dispositions *D*_1_,...,*D*_*n*_ is explained by postulating a common cause $\mathcal {D}$.

In the next section we take up the idea of combining creative abduction and principles of causation by modeling cases of successful creative abduction in a Bayes net framework. Though Bayes nets can be causally interpreted, one does not have to subscribe to a realist interpretation when employing this particular framework to model creative abduction. While the realist gets a justification for creative abductive inferences on the basis of a causal interpretation, the instrumentalist can still use the Bayes net framework without a causal interpretation as a tool for justifying abductive inferences in terms of unificatory power. In this paper we prefer to stay neutral on the realist vs. instrumentalist question. As we will show, modeling creative abduction Bayesian style comes with a couple of advantages regardless of the answer to that question.

## Modeling creative abduction Bayesian style

We start this section by briefly introducing the basics of the Bayes net formalism. Bayes nets allow for modeling and graphically representing the paths over which probabilistic information spreads between variables. A Bayes net consists of a set **V** of random variables *X*_1_,...,*X*_*n*_, a set **E** of directed edges (→) connecting some of these variables, and a probability distribution *P* over **V**. A triple 〈**V**,**E**,*P*〉 is a Bayes net if and only if it conforms to the Markov factorization (Pearl 2000, p. 16)

6 $$ P(X_{1},...,X_{n})=\prod\limits_{i = 1}^{n} P(X_{i}|\mathbf{Par}(X_{i})), $$

where **Par**(*X*_*i*_) is the set of *X*_*i*_’s parents in the Bayes net’s graph **G** = 〈**V**,**E**〉, i.e., the set of all *X*_*j*_ ∈**V** for which *X*_*j*_→*X*_*i*_ holds. Whenever the probability distribution *P* of a triple 〈**V**,**E**,*P*〉 factors according to Eq. 6, then one can read off certain independencies in *P* from the graph **G** = 〈**V**,**E**〉. Every *X*_*i*_ ∈**V** has, for example, to be independent of every *X*_*j*_ that is not connected to *X*_*i*_ via a path *X*_*i*_→...→*X*_*j*_ conditional on **Par**(*X*_*i*_). In the causal interpretation, the arrows (→) of a Bayes net’s graph stand for direct cause-effect relationships. It is well-known that **(CCP)** is a consequence of assuming the causally interpretated Markov factorization. Note that Schurz (2008, 2016) only refers to the causal Bayes net framework in order to justify **(CCP)** in support for a realist interpretation of creative abduction.^3^ In contrast, we employ Bayes nets in order to analyze successful instances of creative abduction.

**Fig. 1:**
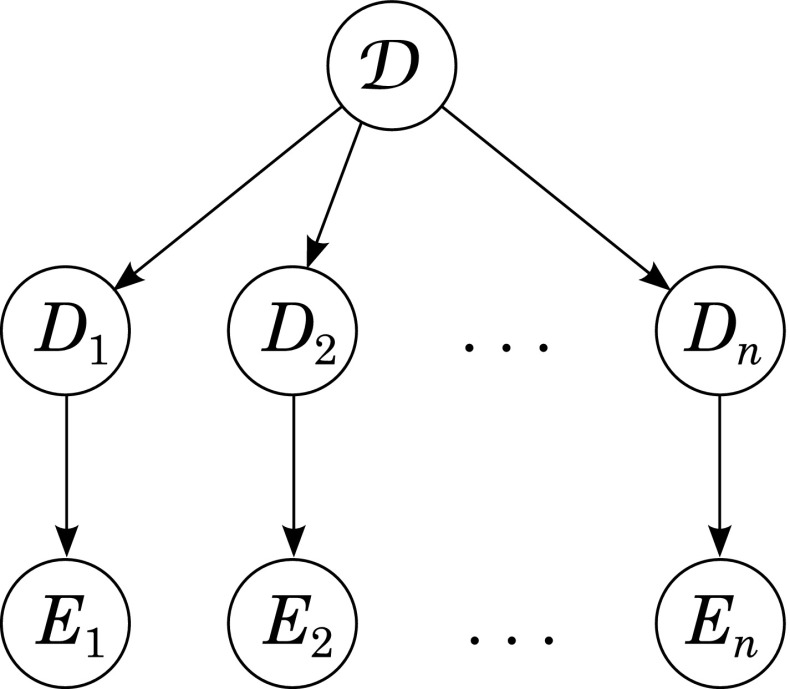
Bayes net for modeling successful instances of creative abduction

Let us now come to the question of how to model successful cases of creative abduction in the Bayes net framework. We represent pairwise empirically correlated lower-level dispositions by variables *D*_1_,...,*D*_*n*_ and the abduced higher-level disposition by a variable $\mathcal {D}$. Evidence for one of the lower-level dispositions *D*_*i*_ (with 1 ≤ *i* ≤ *n*) is represented by a variable *E*_*i*_ which stands for an inductive generalization of instances of test-reaction conditions such as (*T*_*i*_(*a*_1_,*t*_1_) ∧ *R*_*i*_(*a*_1_,*t*_1_)) ∧ ... ∧ (*T*_*i*_(*a*_*k*_,*t*_*l*_) ∧ *R*_*i*_(*a*_*k*_,*t*_*l*_)). The dependence of each lower-level disposition *D*_*i*_ on its corresponding evidence *E*_*i*_ is represented the same way as the dependence of a hypothesis on its evidence is typically modeled in the Bayesian framework: For each pair *D*_*i*_,*E*_*i*_ we draw an arrow *D*_*i*_→*E*_*i*_. Since the creative abductive step is conducted by applying **(CCP)** in Schurz’ (2008) original approach, we introduce the higher-level disposition variable $\mathcal {D}$ as a common parent of the lower-level disposition variables *D*_1_,...,*D*_*n*_. The resulting graph is depicted in Fig. 1.

Probability flow between dispositions *D*_1_,...,*D*_*n*_ is established via $\mathcal {D}$ if the following general conditions are satisfied:

1. $\mathcal {D}$ is not extreme, i.e., $0<P(\mathcal {D})<1$.
2. Each *D*_*i*_ depends positively on $\mathcal {D}$, i.e., $P(D_{i}|\mathcal {D})>P(D_{i})$.

From 1. and 2. it follows that *P*(*D*_*i*_|*D*_*j*_) > *P*(*D*_*i*_) if *i*≠*j*. (For a proof see, e.g., Dardashti et al. 2017.) To account for the corresponding correlations between the evidence *E*_1_,...,*E*_*n*_, the following condition has to be satisfied as well:

3. Each *E*_*i*_ depends positively on its corresponding *D*_*i*_, i.e., *P*(*E*_*i*_|*D*_*i*_) > *P*(*E*_*i*_).

From 1., 2., and 3. it follows that *P*(*E*_*i*_|*E*_*j*_) > *P*(*E*_*i*_) if *i*≠*j*.

Conditions 1., 2., and 3. are necessary conditions for successful creative abduction: They guarantee pairwise correlations among lower-level dispositions that have to be inductively inferred on the basis of observed evidence and build the basis for introducing the higher-level disposition $\mathcal {D}$ which is then, in turn, used to explain these correlations.^4^

Like in Schurz’ (2008) original approach, creative abduction provides unification if modeled Bayesian style. In the original approach (see Section 2) introducing the higher-level disposition $\mathcal {D}$ provided unification of *n*^2^ empirical law statements establishing pairwise empirical correlations among *n* lower-level dispositions to *n* higher-level dispositional statements. In the Bayes net setting, pairwise empirical correlations between *n* lower-level dispositions *D*_1_,...,*D*_*n*_ consist in $\binom {n}{2}$ probabilistic dependencies of the form *P*(*D*_*i*_|*D*_*j*_) > *P*(*D*_*i*_), where 1 ≤ *i*≠*j* ≤ *n*. Similarly, for the dependencies among pairs of evidential variables there are $\binom {n}{2}$ empirical correlation statements of the form

7 $$ P(E_{i}|E_{j})>P(E_{i})\text{, where \(1\leq i\not=j\leq n\).} $$

It follows from the Markov factorization (Eq. 6) that these $\binom {n}{2}$ empirical correlation statements can be unified by the 2*n* + 1 probabilistic statements in conditions 1., 2. and 3.: *n* statements of the form *P*(*E*_*i*_|*D*_*i*_) > *P*(*E*_*i*_) (with 1 ≤ *i* ≤ *n*), *n* statements of the form $P(D_{i}|\mathcal {D})>P(D_{i})$ (with 1 ≤ *i* ≤ *n*), and 1 statement $0<P(\mathcal {D})<1$. To compare Schurz’ (2008) approach and the Bayesian approach w.r.t. their unificatory power, we introduce a simple measure *u* intended to capture the intuitions about unification outlined above. Given *n* correlated lower-level dispositions, *u*(*n*) measures the ratio between *x*(*n*) empirical statements to be unified and *y*(*n*) unifying theoretical statements. In order to shift the neutral case to 0, we subtract 1 from this ratio: $u(n)=\frac {x(n)}{y(n)}-1$. Its output is in the interval [− 1,*∞*), where a negative value means that the theoretical description is more costly than simply listing the empirical statements, 0 means that there is no gain but also no cost in providing a theoretical description, and a positive value means that the theoretical description provides unification.^5^

**Fig. 2:**
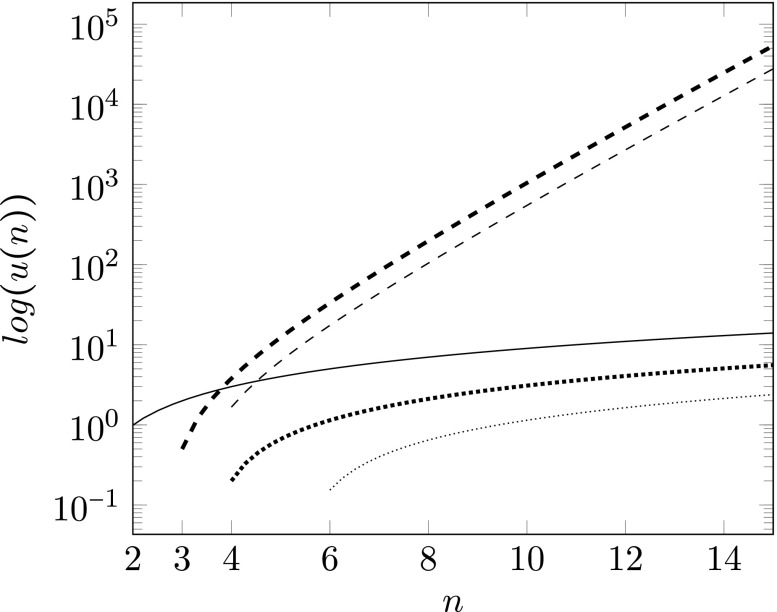
Comparison of unificatory power in the original and in the Bayesian setting: *n* is the number of pairwise empirically correlated dispositions. *u*(*n*) measures the unificatory power given *n* such dispositions by taking the ratio between the number of their corresponding empirical law statements and the number of unifying statements with a shift of the neutral case to 0. In the original setting (thin solid line), *u*(*n*) is calculated via $\frac {n^{2}}{n}-1$, where *n*^2^ is the number of empirical law statements in Eq. 4. The unifying statements consist of the *n* formulae in Eq. 5. In the Bayesian setting (thin dotted line), the corresponding *u*(*n*) is calculated via $\frac {\binom {n}{2}}{2n + 1}-1$. The nominator $\binom {n}{2}$ expresses the number of statements describing the strict (unconditional) empirical correlations in Eq. 7, and the denominator 2*n* + 1 is the number of unifying statements in conditions 1., 2., and 3. Omitting the lower-level dispositions *D*_1_,...,*D*_*n*_ results in a slight boost of unificatory power (thick dotted line): If one operates directly with the higher-level disposition $\mathcal {D}$, *u*(*n*) is calculated via $\frac {\binom {n}{2}}{n + 1}-1$. Again, $\binom {n}{2}$ expresses the number of statements describing the empirical correlations, and *n* + 1 is the number of unifying statements (condition 1 and *n* statements of the form $P(E_{i}|\mathcal {D})>P(E_{i})$, where 1 ≤ *i* ≤ *n*). The unificatory power *u*(*n*) in the Bayesian setting with conditional dependencies (thin dashed line) is calculated via $\frac {2^{n-2}\cdot \binom {n}{2}}{2n + 1}-1$. The numerator expresses the number of statements describing the conditional and unconditional dependencies according to Eq. 8, and the denominator 2*n* + 1 is, again, the number of unifying statements in conditions 1., 2., and 3. When directly operating with $\mathcal {D}$ in this setting (thick dashd line), again, a boost in unificatory power results. In this setting *u*(*n*) is calculated via $\frac {2^{n-2}\cdot \binom {n}{2}}{n + 1}-1$. The latter two cases show that once one allows for non-strict (conditional) correlations, then abductive inference in the Bayes net setting receives a tremendous boost in terms of unificatory power. Note that the y-axis plots the logarithm of the ratio with a shift of the neutral case to 0

A comparison of the unificatory power of both, the original and the Bayes net approach, is provided in Fig. 2 (thin solid line and thin dotted line): In the case of strict (unconditional) correlations, the original approach fares better than the Bayesian approach. This is due to the theoretical power of the Bayesian framework which requires more parametrization. However, one can increase the performance of the Bayesian approach (see thin and thick dotted line in Fig. 2) by omitting the intermediate lower-level dispositions *D*_1_,...,*D*_*n*_ in the 2*n* + 1 statements used for unifying the correlations among the evidence *E*_1_,...,*E*_*n*_ and explain these correlations directly by *n* statements of the form $P(E_{i}|\mathcal {D})>P(E_{i})$ (with 1 ≤ *i* ≤ *n*) and 1 statement $0<\mathcal {D}<1$ instead.^6^ While introducing the lower-level dispositions *D*_1_,...,*D*_*n*_ might be practically necessary to find a more general higher-level disposition $\mathcal {D}$, the presence of these lower-level dispositions should not be counted against the unificatory value of the larger theory since all the theoretical gain achieved by the unification can eventually be traced back to the presence of the higher-level disposition $\mathcal {D}$.^7^

Up to now we focused on comparing the unification of statements about *unconditional* empirical correlations. However, many more empirical correlations are possible in the Bayesian setting. If the evidential base is strictly correlated (i.e., *P*(*E*_*i*_|*E*_*j*_) and $P(E_{i}|\overline {E}_{j})$ with 1 ≤ *i*,*j* ≤ *n* are extreme), then it follows from Eq. 6 and conditions 1., 2., and 3. that each two variables *E*_*i*_,*E*_*j*_ (with *i*≠*j*) are independent conditional on any set of other evidential variables. Thus, the unconditional dependence statements in Eq. 7 capture all dependencies among variables *E*_1_,...,*E*_*n*_ in this setting. However, if some correlations among pieces of evidence cannot be screened off by some non-empty set of other evidential variables, then also many *conditional* empirical dependencies may hold among pairs of evidential variables. In particular, there can be up to $2^{n-2}\cdot \binom {n}{2}$ empirical dependencies of the form

8 $$\begin{array}{@{}rcl@{}} &&P(E_{i}|E_{j},\mathbf{Z})>P(E_{i}|\mathbf{Z}),\text{where}\\ &&1\leq i\neq j\leq n \text{and} \mathbf{Z}\subseteq\{E_{k}:1\leq i\neq k\neq j\leq n\}. \end{array} $$

If these conditional dependencies are also taken into account, then creative abduction Bayesian style provides a tremendous gain in unificatory power (see Fig. 2, thin dotted and thin dashed line as well as thick dotted and thick dashed line). From 1., 2., and 3. it also follows that *P*(*E*_*i*_|**Y**) > *P*(*E*_*i*_|**Z**), where **Z** ⊂**Y** and **Y** are sets of evidential variables different from *E*_*i*_. (For a proof see, e.g., Dardashti et al. 2017.) So, the Bayes net framework allows for a much more fine-grained modeling of non-strictly empirically correlated dispositions which can be found in many higher-level sciences such as economics, medicine, psychology, and sociology.

As the comparison in Fig. 2 shows, the original approach proposed by Schurz (2008) and our Bayesian approach perform differently well in different settings. In the case without conditional correlations, the strict approach fares better. It provides more unificatory power and leads already to unification with only two empirically correlated dispositions, while our Bayes net approach requires at least four empirically correlated dispositions to produce positive unificatory power. In the non-strict setting with conditional correlations, on the other hand, Schurz’ approach is not applicable. This is the setting where the Bayesian approach excels. Although the version with 2*n* + 1 unifying statements also requires at least four empirically correlated dispositions to produce positive unificatory power, the amount of unificatory power provided explodes. The version with *n* + 1 unifying statements fares even better. Note that it already provides positive unificatory power with three empiricaly correlated dispositions. These results suggest that the two approaches might rather be seen as complementing each other than as concurring accounts.

## Possible applications and connections to other issues

In this section we outline possible applications of modeling creative abduction Bayesian style and connections to other topics from the philosophy of science literature. In particular, we discuss how abduced theoretical concepts allow for use-novel predictions, how the approach fits with a recent proposal to solve the problem of underdetermination, and how it provides new possibilities for confirmation. Finally, we briefly discuss how results from the causal discovery literature could be used to approach creative abduction from an epistemic perspective.

### Use-novel predictions

Let us illustrate how creative abduction in a Bayes net model allows for generating use-novel predictions^8^ by means of the magnet example introduced in Section 2. Our line of reasoning here is in accordance with Schurz (2008). Although regarding use-novel facts our framework does not add anything to his argumentation, we think that it is good to see that the Bayesian approach can provide use-novel predictions as well. Assume that an empirical correlation between the two dispositions of attracting iron (*D*_1_) and producing electricity when being moved along a wire (*D*_2_) had been established by experimenting with lodestone. It is inferred by abductive inference that this correlation is brought about by the higher-order disposition of generating an electromagnetic field ($\mathcal {D}$). In our approach, this means that one subscribes to a dispositional pattern captured by a Bayes net model with the structure $D_{1}\longleftarrow \mathcal {D}\longrightarrow D_{2}$. Now assume that one finds an object that is not a lodestone, but attracts iron anyway (*D*_1_). It follows from our model together with conditions 1. and 2. that this increases the probability that this object’s having disposition $\mathcal {D}$ brought about its having disposition *D*_1_. Hence, the probability for $\mathcal {D}$ is increased as well. But since $\mathcal {D}$ also increases the probability of this object’s having the disposition to produce electricity by being moved along a wire, also the probability of *D*_2_ is increased. Thus, observing that the object has disposition *D*_1_ predicts that *P*(*D*_2_|*D*_1_) > *P*(*D*_2_) applies to it as well. Note that this prediction is use-novel since only lodestone was used in building the theoretical model.

### Confirmation

Given two dispositions *D*_1_ and *D*_2_ are empirically correlated, it seems to be commonly accepted that one can use evidence for one of these dispositions to confirm the presence of the other disposition. If, for example, one finds that an object attracts iron (*E*_1_), then one tends to accept this as evidence that it has the disposition of producing electricity when being moved along a wire (*D*_2_) as well. So *E*_1_ can be understood as a test for whether an object has disposition *D*_2_. This can be justified by help of our model as follows: Once the model’s structure $E_{1}\longleftarrow D_{1}\longleftarrow \mathcal {D}\longrightarrow D_{2}$ has been established via creative abduction, it follows with condition 3. that observing *E*_1_ increases the probability for the presence of *D*_1_ which, in turn, by conditions 1. and 2. increases the probability of the presence of $\mathcal {D}$. Since $\mathcal {D}$ is a positive factor for bringing about *D*_2_ as well, also the probability for *D*_2_’s presence will be increased. Thus, *P*(*D*_2_|*E*_1_) > *P*(*D*_2_) applies to our object and, according to Bayesian confirmation theory, *E*_1_ confirms *D*_2_.^9^ Below we will see that a qualitative model of such confirmation, which might be considered to be a straightforward application of the theory of creative abduction based on the common cause principle **(CCP)**, has several problems. In this sense, expanding the account by switching to the Bayes net framework seems to allow for increased applicability.

### The problem of underdetermination

This problem arises due to the fact that two different theories or hypotheses *H*_1_ and *H*_2_ can often account for some evidence *E* equally well. So, just considering *E*, it is underdetermined which hypothesis one should choose. One approach to this problem consists in employing indirect evidence *E*^′^ (Laudan and Leplin 1991, p. 464): Assume that *H*_2_, but not *H*_1_ is derivable from a more general theory $\mathcal {H}$, which also entails another hypothesis *H*_3_. Assume further that *E*^′^ is direct evidence for *H*_3_. Now Laudan and Leplin propose that *E*^′^ cannot only be employed for confirming *H*_3_ and $\mathcal {H}$, but also for confirming *H*_2_. Their argument for cashing out *E*^′^ in order to confirm *H*_3_ can be stated as follows (cf. Okasha 1997, pp. 252f):

i $\mathcal {H}$ entails *H*_2_ and *H*_3_ (but not *H*_1_). Furthermore, *E*^′^ confirms *H*_3_.
ii Hence: *E*^′^ confirms also $\mathcal {H}$. (with i)
iii Hence: *E*^′^ confirms also *H*_2_. (with i and ii)

However, Okasha (1997) has noted that Laudan and Leplin’s (1991) solution falls victim to problems that arise due to qualitative assumptions about confirmation. The underlying principle which grants the inference from i to ii is the so-called converse consequence condition **(CCC)**:

**(CCC)**: If *A* entails *B* and *C* confirms *B*, then *C* also confirms *A*.

And the underlying principle which grants the inference of iii is the so-called special consequence condition **(SCC)**:

**(SCC)**: If *A* entails *B* and *C* confirms *A*, then *C* also confirms *B*.

Both, **(CCC)** and **(SCC)**, were already discussed by Hempel (1965), who wrote:

“Special Consequence Condition: If an observation report confirms a hypothesis *H*, then it also confirms every consequence of *H*. [… The other condition is] the condition that whatever confirms a given hypothesis also confirms every stronger one. [… This principle might be called] ‘converse consequence condition’.” (Hempel 1965, pp. 31f)

Hempel (1965) also demonstrated that these two principles taken together trivialize the notion of qualitative confirmation because they imply that every statement confirms every other statement. The reason for this is simple:

1. Trivially, *A* entails *A*.
2. Hence, by **(SCC)**: *A* confirms *A*.
3. Trivially also *A* ∧ *B* entails *A*.
4. Hence, by **(CCC)**: *A* confirms *A* ∧ *B*.
5. But then, again by **(SCC)**: *A* confirms *B*.

**Fig. 3:**
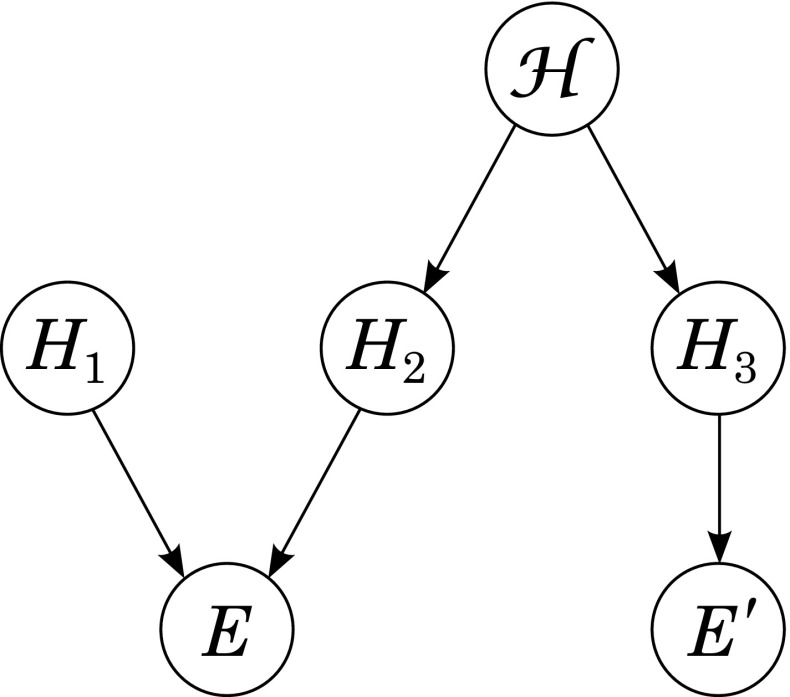
Bayes net modeling Laudan and Leplin’s (1991) solution to the problem of underdetermination

Clearly, this problem does not show up for the (comparative and) quantitative notion of confirmation. If we take, for example, the positive relevance notion of confirmation, then for some *A*,*B*,*C* it is well possible that *Pr*(*A*|*C*) ≤ *Pr*(*A*) (*C* is *not* positively relevant for *A*) though *Pr*(*A*|*B*) > *Pr*(*A*) (*B* is positively relevant for *A*) and *Pr*(*B*|*C*) > *Pr*(*B*) (*C* is positively relevant for *B*). The question arises, how then Laudan and Leplin’s (1991) proposal can be carried out by help of a quantitative notion of confirmation. This is where our probabilistic Bayesian approach to model creative abduction comes into play. We can model Laudan and Leplin’s proposal in a quantitative (probabilistic) way by the Bayes net depicted in Fig. 3. In this model it follows that *E*^′^ confirms *H*_2_, but not *H*_1_: Like in the paragraph about confirmation, *E*^′^ confirms *H*_2_ simply because *P*(*H*_2_|*E*^′^) > *P*(*H*_2_) holds due to conditions 1., 2., and 3.: The mentioned theorem of Dardashti et al. (2017) shows that given these conditions probabilistic flow between *E*^′^ and *H*_2_ is guaranteed, and more generally that positive relevance is transmitted via such paths.^10^ Furthermore, *E*^′^ does not confirm *H*_1_ because *P*(*H*_1_|*E*^′^) = *P*(*H*_1_) holds. This is a direct consequence of the Markov factorization (Eq. 6). In this way our approach can be used to justify a quantitative (probabilistic) reading of Laudan and Leplin’s solution to the problem of underdetermination. The quantitative model allows for avoiding problems a qualitative model of successful creative abduction might have when applied to the problem of underdetermination as outlined here.

### The epistemic challenge: search

In this paper we aimed at *modeling* creative abduction in the Bayes net framework. To this end we assumed that creative abduction had already been successfully applied. We did not provide an answer to the epistemic question of how and under which conditions creative abduction can be successfully applied in practice. So the epistemic challenge consists in developing reliable methods to abduce unifying dispositions on the basis of empirical data. As Glymour (2018) points out, this problem is tackled in the literature on search of latent variables (see, e.g., Silva et al. 2006; Kummerfeld and Ramsey 2016). Such procedures would, however, require continuous data rather than binary variables as we used them in this paper. So variables should rather represent the strengths of dispositions than simply the presence of such dispositions to get these approaches to work. How exactly such approaches to latent variable search fit with the classical literature on abduction within philosophy of science has to be investigated in future research.

## Conclusion

This paper was about modeling successful cases of creative abduction on the basis of empirically correlated dispositions within a Bayes net framework. After introducing Schurz’ (2008) strict approach in Section 2, we developed a Bayes net representation of instances of successful creative abduction in the sense of Schurz in Section 3. This move allows for a more fine-grained investigation of the unificatory power gained by creative abduction. It also allows for identifying the relevant necessary conditions for successful cases of creative abduction. Note that our approach to creative abduction can, in a very limited way, be used for purposes of selective abduction as well. It suggests to penalize all dispositions of a given set of candidates that do not meet the necessary conditions for successful creative abduction, i.e., all those $\mathcal {D}$s that (i) are not positively correlated with one of the lower-level dispositions *D*_1_,...,*D*_*n*_ (or one of the pieces of evidence *E*_1_,...,*E*_*n*_) to be explained or (ii) do not screen off all non-intersecting sets of lower-level dispositions (or pieces of evidence) from each other. If (i) were the case, then $\mathcal {D}$ would not explain every lower-level disposition (or piece of evidence), and if (ii) were not the case, the Markov condition would be violated and $\mathcal {D}$ would not fully explain some correlations among lower-level dispositions (or pieces of evidence). In both cases, there might be a better dispositional explanation available. The approach does, however, not come with a criterion for how to select the best disposition(s) $\mathcal {D}$ of a set of rivals all satisfying these necessary conditions. For this purpose, one could use one of the approaches to selective abduction already on the market (see, e.g., Lipton 2004; Niiniluoto 1999; Williamson 2016).

In Section 4 we then discussed several possible applications of modeling creative abduction Bayesian style. In particular, we spelled out how creative abductive inferences can generate use-novel predictions in our setting. We also presented a new possibility to apply Bayesian confirmation theory: Once a higher-level connection between lower-level dispositions has been established via creative abduction, one can confirm the presence of one of these lower-level dispositions by finding evidence for one of the other lower-level dispositions. Another result was that a quantitative (probabilistic) reading of Laudan and Leplin’s (1991) proposed solution to the problem of underdetermination can be supported once one is able to unify one of the competing hypotheses with an additional hypothesis via creative abduction.

This paper was about modeling successful instances of creative abduction and about which interesting conclusions one can draw from a Bayes net representation. An issue that has not been tackled is the epistemic question of how exactly theoretical concepts should be abduced on the basis of empirical data. If dispositions can be adequately represented by continuous variables, then this seems to open the door for a fruitful application of much more sophisticated search procedures from the literature on causal discovery.

## Footnotes

1 Selective abduction is often subsumed under the term *inference to the best explanation*.

2 For more modern analyses of dispositions, see, for example, Lewis (1997), Malzkorn (2000), and Manley and Wasserman (2008).

3 For an argument supporting a realist interpretation of the causal Bayes net framework, see Gebharter (2017) and Schurz and Gebharter (2016).

4 Note that our Bayes net account differs from Schurz’ (2015) approach to unify statistical dependencies and independencies by causal structure. While Schurz reduces a number of statistical dependencies and independencies to a (smaller) number of causal relations, our account reduces a number of correlations among different pieces of evidence to a number of statements postulating abduced dispositions.

5 Measuring unificatory power by counting statements, argument patterns, etc. is common in the unification literature (cf. Woodward 2017, sec. 5.4). There are, however, also other ways of measuring unificatory power. To avoid problems Bayesian measures have with common cause structures (cf. Schupbach 2005), Myrvold (2017) suggests to avoid an explicit representation of common causes. For purposes of unification, one should use hypotheses postulating such common causes instead. But since we focus on creative abduction in this paper, avoiding common causes in order to maintain a Bayesian measure for unification seems to be inappropriate for our endeavor. For this reason and in order to compare the Bayes net analysis with Schurz’ (2008) approach, we decided in favor of a simple counting measure.

6 The conditional probabilities $P(E_{i}|\mathcal {D})$ can be computed as $P(E_{i}|D_{i},\mathcal {D})\cdot P(D_{i}|\mathcal {D})+P(E_{i}|\overline {D}_{i},\mathcal {D})\cdot P(\overline {D}_{i}|\mathcal {D})$.

7 We are indebted to an anonymous referee for pointing this out to us.

8 A prediction is use-novel if it predicts an empirical phenomenon that was unknown at the time of the prediction or that has not been used as evidence in constructing the theory on whose basis this phenomenon is predicted (see, e.g., Worrall 1985, 2006). The ability to produce use-novel predictions is often regarded as a requirement for empirically successful theories since it renders theories independently testable.

9 For a similar line of argumentation in the case of confirmation across analogical systems, see (Dardashti et al. 2017). For possible problems and an extension of this approach, see (Feldbacher-Escamilla and Gebharter ms).

10 We are indebted to an anonymous referee for stressing this parallel between the mentioned intuitions on a qualitative notion of confirmation and the properties of a quantitative notion of confirmation in the Bayesian framework applied here.
